# Acute massive pulmonary thromboembolism caused by cord-like foreign bodies in the heart and pulmonary arteries after percutaneous vertebroplasty: a case report and literature review

**DOI:** 10.3389/fmed.2026.1801254

**Published:** 2026-04-29

**Authors:** Fukang Zou, Yuanyuan Cui, Lefeng Qu, Jianjin Wu

**Affiliations:** 1 Department of Vascular and Endovascular Surgery, The Second Affiliated Hospital of Naval Medical University, Shanghai, China; 2 Department of Radiology, The Second Affiliated Hospital of Naval Medical University, Shanghai, China

**Keywords:** acute pulmonary thromboembolism, cardiopulmonary cement embolism, case report, literature review, percutaneous vertebroplasty

## Abstract

**Background:**

Bone cement leakage is a complication of percutaneous vertebroplasty. However, the cement can extremely rarely extravasate into the heart and pulmonary arteries and form cord-like foreign bodies, inducing acute massive pulmonary thromboembolism.

**Case presentation:**

We present a case of a patient who developed acute massive pulmonary thromboembolism within 2 weeks after PVP, resulting from bone cement leakage into the heart and pulmonary arteries with subsequent thrombosis. Lower extremity vascular ultrasonography ruled out the possibility of thrombus detachment secondary to deep venous thrombosis. Computed tomography pulmonary angiography revealed strip-shaped hypodense filling defects in the main trunks and some branch arteries of the bilateral pulmonary arteries and a cord-like hyperdense shadow. Combined computed tomography pulmonary angiography and three-dimensional reconstruction of the thoracic spine showed that the cord-like foreign body originated from the 8th thoracic vertebra, entered the azygos vein through the vertebral venous system, then continuously traversed the superior vena cava and right cardiac system, and finally extended to the main trunk of the right pulmonary artery, as well as the branches of both pulmonary arteries. Following aggressive surgical thrombolysis and postoperative anticoagulant therapy, the patient’s symptoms immediately improved. Nevertheless, computed tomography pulmonary angiography still showed the cord-like foreign body after 3 months.

**Conclusion:**

Moreover, this report retrospectively analyzes the diagnostic and therapeutic strategies for pulmonary thromboembolism secondary to bone cement leakage after percutaneous vertebroplasty through a literature review.

## Introduction

Cardiopulmonary embolism (CPE) caused by non-thrombotic foreign bodies is extremely rare. Its causes include the retention of iatrogenic foreign bodies, such as central venous catheters and guidewires (1, 2), and residual foreign body fragments from severe trauma, including gunshot wounds or explosive injuries (3). Bone cement leakage after percutaneous vertebroplasty (PVP) can also rarely cause CPE (4, 5).

Bone cement is injected into the collapsed vertebrae during PVP, a surgical procedure for treating conditions such as osteoporosis (6). Cardiopulmonary cement embolism (CPCE) caused by bone cement leakage is extremely rare, with an incidence of only 3.36% (7). Most patients are asymptomatic, and the incidence of symptomatic CPCE is even lower (merely 0.32%) (7). Additionally, the porous structure of bone cement emboli may secondarily induce thrombosis (8). However, to our knowledge, there are almost no reported cases of CPCE-induced acute massive pulmonary thromboembolism combined with secondary thrombosis.

This article reports a case of acute massive pulmonary thromboembolism secondary to cord-like foreign bodies in the heart and lungs after PVP. The source of the foreign body was confirmed using computed tomography pulmonary angiography (CTPA) three-dimensional reconstruction and the patient’s medical history. Emergency thrombolysis was performed, followed by standardized postoperative anticoagulation for 3 months, achieving a favorable outcome.

## Case presentation

A 66-year-old woman presented suddenly with dyspnea of no apparent cause, accompanied by chest tightness and chest pain. She came to the emergency department 3 h after the symptoms had begun. She reported no history of common etiologies associated with acute chest pain, including coronary artery disease, pneumonia, and thoracic trauma. Physical examination revealed coarse breath sounds in both lungs and a hyperactive P2 on cardiac auscultation. She was hemodynamically unstable since her heart rate was 120–130 beats per minute, systolic blood pressure was 85 mmHg, diastolic blood pressure was 54 mmHg, and respiratory rate was 32 breaths per minute.

**FIGURE 1:**
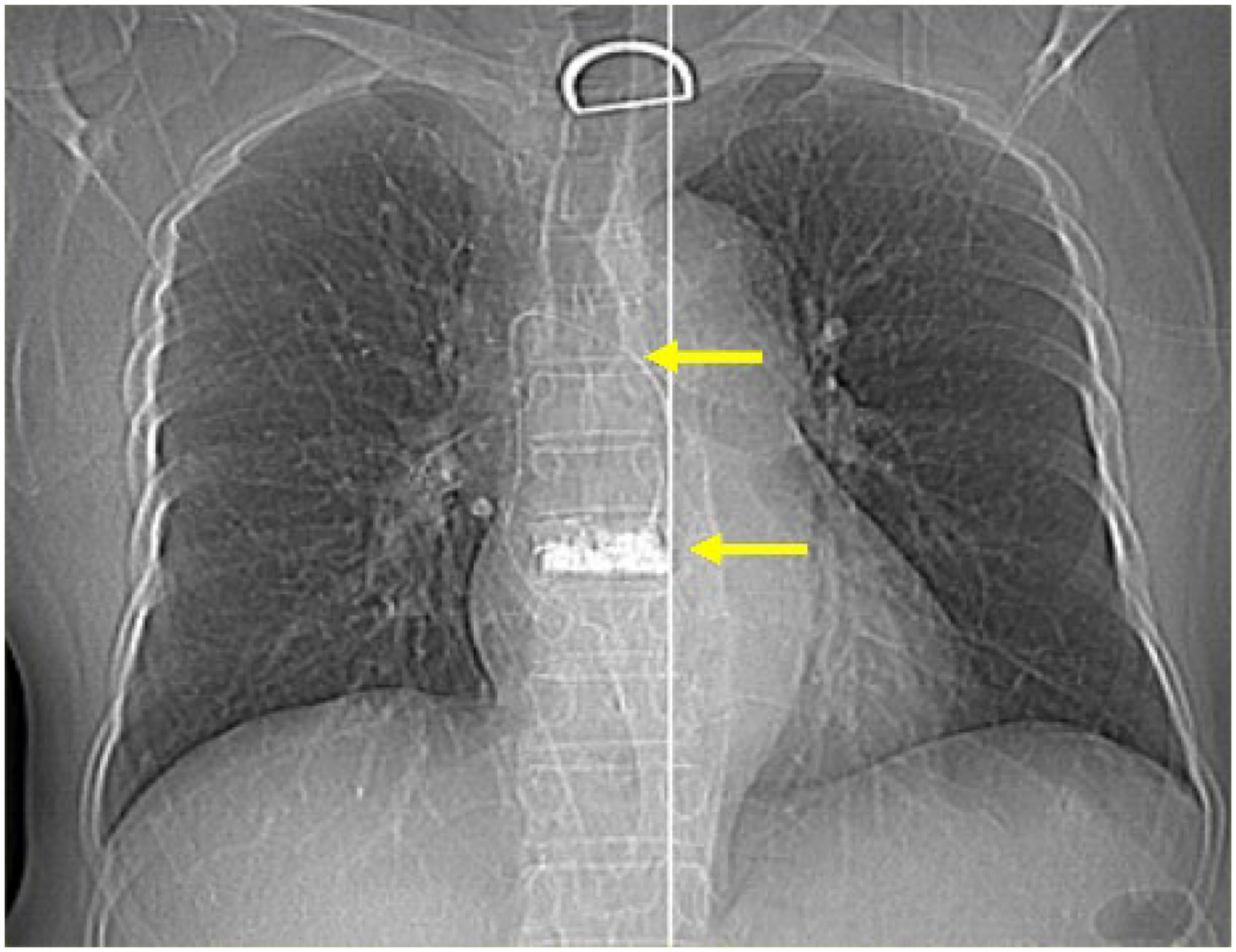
X-ray shows the high-density shadow from the eighth thoracic vertebrae to the right lung (yellow arrows).

**FIGURE 2:**
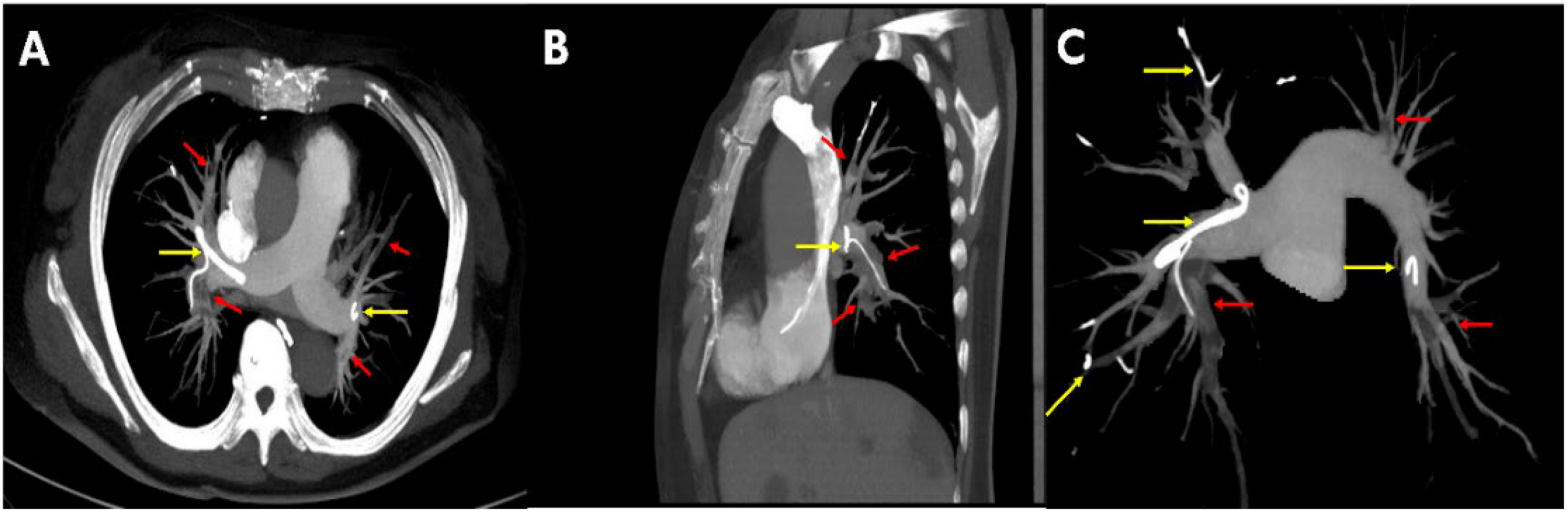
Computed tomography pulmonary angiography (CTPA) showed multiple low-density shadows in the primary and peripheral branches of both pulmonary arteries indicating thromboembolism (red arrows); A high density cord foreign body in the right atrium and pulmonary artery, multiple exuding foreign bodies in both side’s pulmonary branch arteries (yellow arrows). [**(A)** Axial view, **(B)** sagittal view, **(C)** coronal view].

Electrocardiography (ECG) demonstrated an incomplete right bundle branch block. Emergency laboratory investigations revealed markedly elevated D-dimer (33.97 mg/L) and fibrin degradation product (FDP) levels (24.3 mg/L). Following intranasal high-flow oxygen therapy (6 L/min), her oxygen saturation ranged from 85% to 90%. X-ray showed a hyperdense lesion at the T8 vertebral level, alongside a cord-like opacity extending from the T8 vertebra to the right lung field (Figure 1). CTPA indicated multiple thromboembolisms in the primary and peripheral branches of both pulmonary arteries, with more severe lesions on the right side (Figure 2, red arrow). Additionally, a hyperdense cord-like foreign body was identified in the right pulmonary artery, along with multiple scattered foreign body particles in the bilateral pulmonary branch arteries (Figure 2, yellow arrow). The patient was diagnosed with acute massive pulmonary embolism, E1 (persistent hypotension accompanied by cardiogenic shock), but the cause remained unclear.

Color ultrasonography of the lower extremity vessels excluded deep venous thrombosis (DVT) in the lower extremities. Combined three-dimensional (3D) CT reconstruction of the thoracic vertebrae, pulmonary arteries, and heart revealed that the cord-like foreign body originated from the 8th thoracic vertebra. It entered the azygos vein through the vertebral venous system, then continuously traversed the superior vena cava and right cardiac system, and finally extended to the main trunk of the right pulmonary artery and the branches of both pulmonary arteries (Supplementary Video 1). Further inquiry into the patient’s medical history showed no history of vascular-related surgeries or trauma. The patient only sustained a compressive fracture of the vertebral body due to osteoporosis, for which she underwent PVP one week ago. She had no other previous medical history, surgical history, or medication history. The CT characteristics of the cord-like foreign body were consistent with those of the bone cement in the thoracic vertebrae. Combining the medical history and imaging findings, we confirmed that cardiopulmonary embolism was caused by the cord-like foreign body resulting from the extravasation following bone cement injection in the lumbar vertebrae.

After hospitalization, the patient immediately received a subcutaneous injection of 4,000 IU enoxaparin sodium. The patient was diagnosed with grade E1 pulmonary embolism. According to the AHA/ACC Guidelines for the Evaluation and Management of Acute Pulmonary Embolism in Adults, urgent catheter-directed thrombolysis and other treatments are indicated (9). We also obtained consultations from interventional radiology and respiratory medicine, with both recommending primary interventional catheter-directed thrombolysis. Therefore, emergency angiography and pulmonary artery catheter thrombolysis (CDT) were performed, as well as lower extremity venography, to exclude lower extremity DVT again. As pulmonary arteriography is basically the same intraoperative imaging as CTPA, local thrombolysis was conducted (rt-PA 25 mg local perfusion via the catheter), followed by the immediate improvement on imaging. Postoperatively, symptoms (including chest tightness and dyspnea) were alleviated, and her oxygen saturation increased back to 95% (oxygen concentration 2 L/min).

**FIGURE 3:**
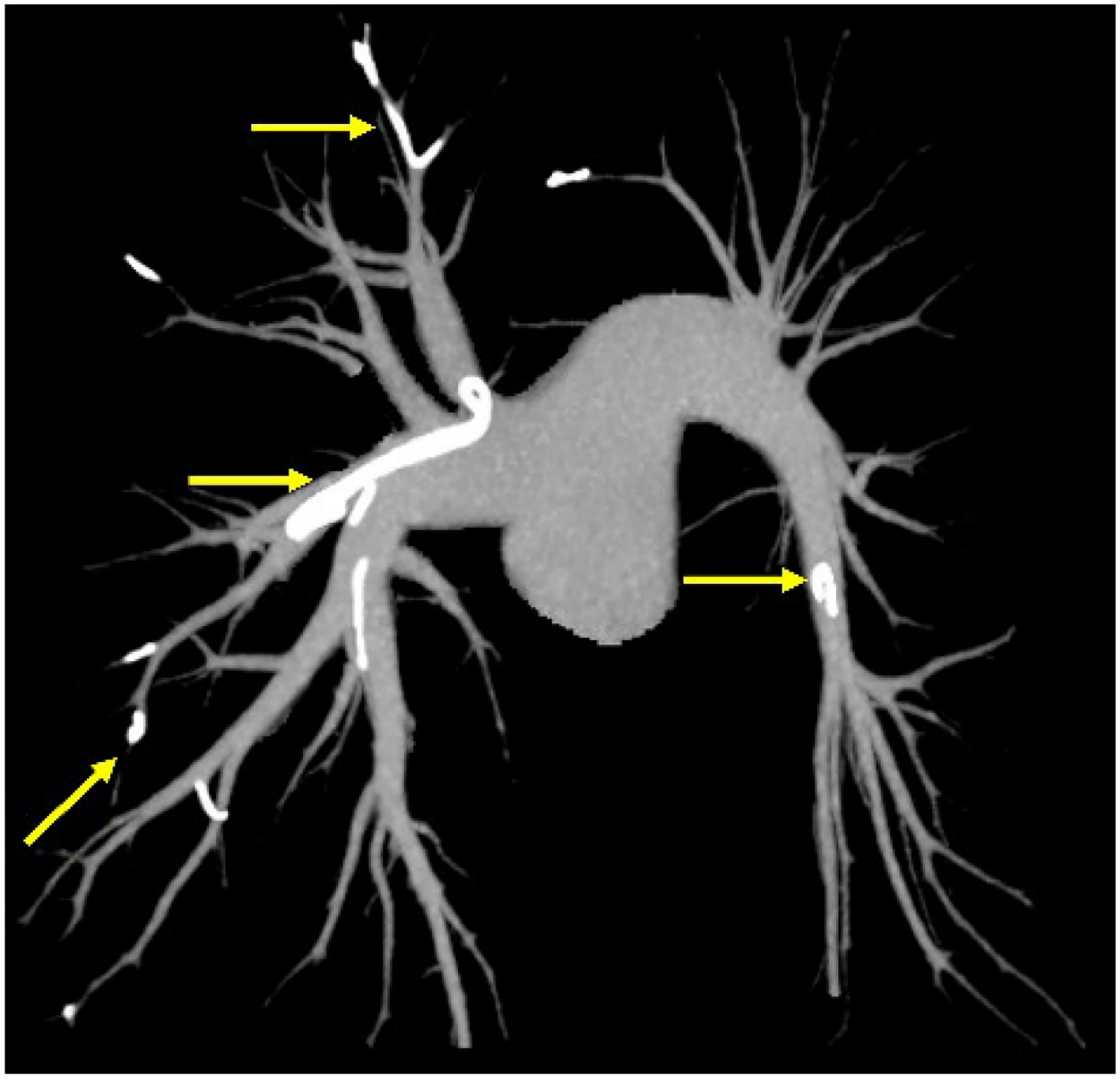
Computed tomography pulmonary angiography (CTPA) of the pulmonary arteries showed that striated dense shadows in the lungs persisted after the patient’s treatment.

The patient recovered well after 3 days, and her discomfort disappeared, while mobility was restored. Then, we proposed to the patient further thoracotomy as a treatment option to remove the bone cement and fully informed the patient of the potential risks of the procedure. As the patient’s symptoms had improved significantly, and she was concerned that thoracotomy might lead to more severe complications, she declined further surgical treatment via thoracotomy. Consequently, only subsequent anticoagulant medical therapy was administered. The patient received enoxaparin sodium 4,000 IU subcutaneously b.i.d. for 1 week and edoxaban 60 mg orally q.d. for 6 months and was discharged from the hospital. After 6 months of formal anticoagulation therapy, CTPA examination was repeated (Figure 3), showing that bilateral pulmonary artery thrombosis basically disappeared, but dense shadows in the proximal branches of the right pulmonary artery trunk, the superior vena cava, the singular vein, and the right atrium still existed. Considering that there was no recurrent pulmonary embolism and no concomitant symptoms related to the foreign body, it was decided that no endoluminal or open surgery could be performed to remove the foreign body, and the patient was instructed to continue with CTPA follow-up annually.

## Discussion

Cardiopulmonary embolism is an extremely dangerous, potentially life-threatening clinical condition. DVT of the lower extremities, with subsequent thrombus detachment and migration to the heart and lungs, is a common cause of CPE. Standardized clinical diagnosis and treatment guidelines are available for such patients (9).

Cardiopulmonary embolism caused by intravascular foreign bodies is extremely rare, with examples including the retention of intravascular catheter devices due to iatrogenic factors, such as central venous catheters, intravenous infusion tubes, guidewires, or vascular sheaths (1, 2). Another cause is residual foreign body fragments from severe trauma, such as gunshot wounds or explosive injuries (3).

Bone cement (polymethylmethacrylate, PMMA) leakage into blood vessels, heart, and lungs after PVP is also one of the rare etiologies of CPE (10). Notably, to our knowledge, there are no reports in the literature of such extensive and continuous cord-like migration of PMMA from the T8 vertebra via the vertebral venous plexus to the superior vena cava, right cardiac system, and main pulmonary artery and its branches.

The incidence of CPCE is only 3.36% in patients with PVP (7). Moreover, most CPCE patients are asymptomatic, and the incidence of symptomatic CPCE is even lower (merely 0.32%) (7). Additionally, PMMA may release MMA, leading to coagulation activation and hemodynamic instability (11); thus, secondary thrombosis may occur. However, to our knowledge, there are almost no reported cases of acute massive pulmonary thromboembolism caused by CPCE complicated with secondary thrombosis.

Polymethylmethacrylate viscosity is an important risk factor for leakage. A lower-viscosity PMMA with longer hardening time is usually selected to better allow the bone cement to integrate into the trabecular bone (12). However, this also increases the risk of PMMA leakage (13). Furthermore, several factors increase the risk of PMMA leakage into the heart and lungs: the involvement of thoracic vertebrae, higher cement injection volume per segment, treatment of >3 vertebrae per case, and venous cement leakage (14–16).

Although most patients with CPCE are asymptomatic (7), PMMA may release MMA, leading to coagulation activation and hemodynamic instability. This may further induce thrombosis (8). Therefore, asymptomatic CPCE patients still require sufficient attention and standardized clinical anticoagulant management. Symptomatic clinical manifestations include chest pain, dyspnea, and acute respiratory distress syndrome (ARDS), which may be fatal (17).

To date, there is no consensus or guideline for treating patients with CPCE. For asymptomatic patients, management focuses on early diagnosis and regular follow-up, and active clinical intervention is usually not required. The literature indicates that CTPA has high sensitivity for diagnosing CPCE (18). Based on the present case, CTPA combined with 3D reconstruction of the thoracic spine CT may be considered a valuable imaging tool for the early detection of postoperative CPCE in symptomatic patients following PVP.

For patients with a history of PVP who present with chest tightness or chest pain, physicians should be highly vigilant about the possibility of CPCE. Thus, immediately performing CTPA combined with 3D reconstruction of the relevant thoracic spine CT is recommended to confirm the presence of bone cement embolism and simultaneously check for secondary thrombosis. Based on the experience of this case and relevant literature, anticoagulant therapy should be initiated immediately after confirmation (19–21). If necessary, surgical thrombectomy may be considered (4, 22, 23).

## Data Availability

The original contributions presented in this study are included in the article/Supplementary materials, further inquiries can be directed to the corresponding authors.

## Supplementary material

Supplementary Video 1 Combined three-dimensional (3D) CT reconstruction of the thoracic vertebrae, heart and pulmonary arteries.
